# Zika virus endemic challenges during COVID-19 pandemic in Africa

**DOI:** 10.1186/s41182-021-00372-6

**Published:** 2021-10-13

**Authors:** Abdullahi Tunde Aborode, Mahnoor Sukaina, Harendra Kumar, Tahreem Farooqui, Samar Faheem, Priyanka Chahal, Luay Alkazmi, Helal F. Hetta, Gaber El-Saber Batiha

**Affiliations:** 1 Health Africans Platform, Research and Development, Ibadan, Nigeria; 2 West African Academy of Public Health, Research and Development, Abuja, Nigeria; 3 Karachi Medical and Dental College, Karachi, Pakistan; 4 Dow University of Health Sciences, Karachi, Pakistan; 5 S. Tentishev Asian Medical Institute, Kant, Kyrgyzstan; 6 Department of Biology, Faculty of Applied Sciences, Umm Al-Qura University, Makkah, 21955 Saudi Arabia; 7 Department of Medical Microbiology and Immunology, Faculty of Medicine, Assiut University, Assiut, 71515 Egypt; 8 Department of Internal Medicine, University of Cincinnati College of Medicine, Cincinnati, OH 45267-0595 USA; 9 Department of Pharmacology and Toxicology, Faculty of Veterinary Medicine, Damanhour University, Damanhour, Egypt

**Keywords:** Zika virus, Endemic, COVID-19 pandemic, Challenges, Africa

## Abstract

Zika virus remains endemic and opportunistic of high transmission in the tropical region of Africa, and the repeated cases of the Zika virus in Africa made it public health emergency in 2016. Amidst the COVID-19 pandemic, the catastrophic cases of unknown and unreported deaths overwhelming the region of Africa could not give health attention to respond to other endemic diseases. Here, we present the possible complication and challenges associated with the Zika virus in Africa and COVID-19 predominance, shifting the attention from the Zika virus surveillance. This paper determines to enlighten the reader about the situation, the efforts to curb the transmission of both the Zika virus and the COVID-19 pandemic. Therefore, the report recommends sustainable solutions that can lessen the threat to public health.

## Dear Editor,

Zika virus was identified first in 1947 in Entebbe Uganda Forest, which was found in an isolated Aedes mosquito [1, 2]. In 1952, the Zika virus was discovered in humans and confirmed that humans could spread it, as reported in 1962. Later in 1963, it was identified in Uganda [2]. Following that global health emergency was declared due to the Zika virus outbreak in 2016 [1]. The viral infection stayed for 60 years in Africa, originating from the East then spread to the West following Central and North Africa. In 2016, there was a widespread Zika virus infection outbreak in the West African region of Cabo Verde with 7,000 cases of Aedes species infection in humans [1].

The Zika virus originated from Africa transmitted infection that outreached Brazil in 2015, and the virus prevalence still causes problems in Brazil amidst COVID-19 in Brazil [2, 3]. This reported case extends the cases of neurological disorders by which WHO declared Public Health Emergency on international Concern (PHEIC) on February 1, 2016 [2].

However, the growth of the Zika virus in Africa has spread to different parts of African countries through other routes, and it is possible due to common diseases surveillance. It has become more challenging for most African countries to track down those infected, resulting in undiagnosed death rates in Africa. Therefore, this situation could be a looming case to deal with, especially during the COVID-19 pandemic [4].

Moreover, while African countries are working towards overcoming the arbovirus infection, the global pandemic took a catastrophic turn to the current challenges. According to the WHO, the first peak wave of the COVID-19 pandemic in Africa was reported in July 2020, with a contemporary approximation of more than 120,000 cases [5]. Following the recent situation of COVID-19 in Africa, it is going uphill as the expected third wave of novel COVID-19 has been on the surge, which has spread to 23 countries in the African continent of which Uganda, Nigeria, DRC, Zambia, Namibia, Tunisia, and Rwanda are affected severely by the new delta variant. The delta variant of the COVID-19 pandemic has the potential to transmit faster and is more infectious as it spreads [6].

The WHO has labeled the "worst pandemic week ever" at the end of July 4, 2021, as the cases have exponentially risen to 251,000 in Africa. The mortality rate due to the COVID-19 pandemic in Africa is 2.6%, which is higher than 2.2% of globally affected countries, thus increasing the vulnerability of the African population [6].

According to the World Health Organization, on 14 July 2021, 30,154 new COVID-19 cases and 443,472 active cases have been reported in Africa following 104,924 increasing deaths and 683 new cases of mortality in the region [6]. When it comes to the Zika virus, Africa has faced several challenges. The geographic distribution of mosquito that transmits Zika virus globally coincides with the area of dengue transmission.

The coalition in the clinical picture of the Zika virus and COVID-19 pandemic may increase the probability of misdiagnosis of the diseases. The over-rising in the cases of COVID-19 leads to the low diagnosis of the Zika virus. This situation may cause a delay in the correct diagnosis and worsen the symptoms as the delay in the initiation of treatment predispose. There are some clinical similarities and relationships between the Zika virus and the COVID-19 pandemic; these are explained in detail in Table 1 below.

**Table 1:** The clinical correlation of both Zika Virus and COVID-19

Signs and symptoms	Zika virus	COVID-19
Similar symptoms	Fever, headache, arthralgia, myalgia, non-purulent conjunctivitis, myalgia, rash, nausea, emesis, and abdominal pain Less common symptoms: sore throat, and cough [19]	Fever, headache, arthralgia, myalgia sore throat, and cough Less common symptoms: conjunctivitis, rash, nausea, emesis, and abdominal pain [19]
Dissimilar clinical signs and symptoms	Pruritis, expectorations, chest pain, congenital microcephaly, and Guillain–Barre syndrome [19]	Dyspnea, fatigue, anosmia, ageusia, and diarrhea [19]

On the other hand, *Ae albopictus* generally prefers humans and can be found feeding indoors [7]. *Ae. albopictus* succeeded in colonizing temperate zones such as the United States and Europe. They invaded African countries where it now acts as the primary vector of Dengue virus and chikungunya virus (CHIKV) in urban and rural settings [7, 8]. Among the challenge of the Zika virus amidst the COVID-19 pandemic is the poor disease surveillance and low diagnosis in resource-constrained environments, especially in Africa is enormous [8, 9].

Zika virus diagnosis with a reverse transcription-polymerase chain reaction (RT-PCR) has the low technical expertise to operate it, especially during COVID-19 due to shortage of medical staff to interpret with caution based on the sample used an interval of symptoms onset and date of specimen collection [8]. Another challenge Zika virus poses during the COVID-19 pandemic is the shortage of laboratories to test for the virus because most of the laboratories available have been converted to test for COVID-19 pandemic only and might increase the higher virulence of the Zika virus, if necessary steps are not taken.

Interestingly, now no medications or vaccines are available since Zika presents asymptomatically or with mild symptoms; symptoms may be controlled with bed rest, intravenous fluids, and acetaminophen, making it hard to control and given priority during the COVID-19 pandemic [8]. The major challenge of the Zika virus is the complication of infection, and effort should be placed on antiviral agents, vaccines, and other preventive measures. About 30 FDA, approved antiviral agents have been studied and have significant anti-Zika viral activity [10, 11].

About 56% of Africa's urban population is overcrowded, and only 34% of the continent has access to proper hand-washing facilities [10, 11]. In terms of healthcare, the African continent has insufficient professionals and a lack of hospital beds [10–12]. African countries are not prepared to deal with health emergencies due to their poor infrastructure and inadequate healthcare [12].

Poor road infrastructure and lack of transport facilities make it difficult for people to travel to testing sites. There is a possibility that the number of positive cases is under-reported in the African continent. These drawbacks are amplified when it comes to administering COVID-19 vaccines and giving priority to the Zika virus. Africa has always been a hotspot for different viral diseases, mainly due to poverty, lack of proper medical facilities, untrained health workers, and overpopulation.

Moreover, the existence of the Zika virus in the region with new strains of emerging COVID-19 pandemic cases is not only a burden to health facilities. Still, it is a reason for the increase in mortality in the region. Most of the viruses share similar clinical manifestations, which is a possible cause of ineffective and proper treatment with unfavorable clinical outcomes and poor prognosis [13]. On top of that, the Zika virus, including many other viruses, exists in underdeveloped or developing countries like African countries.

Due to financial constraints affecting African countries, there is limited research in developing effective treatment and management plans. For this reason, it is tough to eradicate these diseases. In all this, the only ray of hope is financial dependence and support from foreign aids, developed countries, and international bodies like WHO. Meanwhile, political instability in most African countries is another reason why there is poor health infrastructure in the region.

For the past couple of decades, the African continent has been an area of serious concern in coping with different epidemics in the region. However, international bodies like WHO, with its volunteer network, are working tirelessly to help foreign governments in the African continent to come out of any unwanted situation. In the prior years of the Zika virus outbreak, it was thought of as restricted to the Americas, but the virus has spread to the islands of Cape Verde, off the coast of Africa. Africa border is volatile and weak, this in turn influence how viruses like the Zika virus, COVID-19 pandemic are spreading in various part of Africa [14].

Africa CDC has prioritized educating the masses about the general preventive measures, including preventing mosquito bites and preventing sexual transmission. Part of the measures created by the healthcare system is preventing mosquito bites by wearing long sleeve shirts and long dresses, permethrin-impregnated clothes, indoor residual spraying of insecticide, screening of doors and windows against mosquitoes, and other environmental control measures aimed at decreasing the breeding of mosquitoes [15].

When it comes to the COVID-19 pandemic, Africa has faced several challenges. The continent is especially vulnerable to disease outbreaks, especially during the COVID-19 pandemic; the disease outbreaks include Ebola, viral hepatitis, yellow fever, etc. [16–18]. However, with all these efforts implementing on Zika virus amidst the COVID-19 pandemic, there is a need for regular public awareness campaigns about preventing mosquito bites through wearing long sleeves and using repellents and mosquito nets. It is also essential to target mosquito-breeding areas.

The public should be warned about leaving water containers lying around, and tanks and sewage areas need to be covered. Insecticides need to be used around the house. Pregnant women and those of childbearing age should also be warned about traveling to places where the Zika virus is endemic. It is important not to overuse insecticides in the fight against Zika and other mosquito-borne diseases to prevent insecticide resistance. However, when using them is inevitable, there are ways to limit the growth of resistance.

Different insecticides of different classes and modes of action should be used in rotation instead of one insecticide. Interventions should also be combined when fighting an outbreak, i.e., both adult and larvae should be targeted, again with different insecticides. Another option is that when one compound is used in an area, a different one should be used in its neighboring regions. To implement these strategies in an outbreak, it is essential to have experts who know insecticide products and resistance mechanisms.

Monitoring the mosquito population and its insecticide resistance is crucial on a large scale to prevent a Zika virus outbreak. Constant surveillance of resistance within the mosquito population is necessary to anticipate future outbreaks. A way to achieve this is if governments of developing countries work together with WHO to monitor the mosquito population.

When it comes to the constantly evolving COVID-19 situation, much needs to be done. Governments need to allocate funds to deal with the pandemic effectively. Vaccination needs to be scaled up, as it is the only way to prevent new strains from mutating. Governments need to start allocating funds for booster doses required in the future as new strains emerge and vaccine immunity vanes.

A proper online system for managing every citizen's dose schedule and tracking should be implemented, as soon as possible to keep track so non-compliant individuals with booster shots in the future can also be traced. Public service campaigns should be run on TVs and radios, and misinformation spread on social media should be addressed and disproved.

Government officials should also look into fines or reducing access to public services for people who refuse to get vaccinated, such as reduced access to public transport. Stricter measures need to be taken throughout the continent to implement social distancing and masks, with fines in case of noncompliance.

Free masks should be provided outside buildings before entry, and campaigns should be run emphasizing the correct mask-wearing method. Sanitizers should be delivered to areas where hand-washing facilities are not available.

Overcrowding should be discouraged in indoor spaces, and offices and workplaces should not allow more than necessary employees to come to work. Working and studying from home should be adopted as part of the everyday lifestyle wherever possible. It is also crucial to discourage unnecessary travel and activities where lockdown measures cannot be implemented on a rigorous level. This will allow health practitioners and regulating bodies to design policies effective enough to cope with similar diseases. Conclusively, there is an inevitable need for further researches to understand better the correlation of such conditions with struggling health setups.

## Data Availability

Not applicable.
